# Comparison of hypobaric hypoxia symptoms between a recalled exposure and a current exposure

**DOI:** 10.1371/journal.pone.0239194

**Published:** 2020-09-23

**Authors:** Min-Yu Tu, Kwo-Tsao Chiang, Chao-Chien Cheng, Fang-Ling Li, Yu-His Wen, Sing-Hong Lin, Chung-Yu Lai

**Affiliations:** 1 Aviation Physiology Research Laboratory, Kaohsiung Armed Forces General Hospital Gangshan Branch, Kaohsiung City, Taiwan; 2 Department of Health Business Administration, Meiho University, Pingtung County, Taiwan; 3 Department of Life Sciences and PhD Program in Translational Medicine, National Chung Hsing University, Taichung City, Taiwan; 4 Institute of Medical Science and Technology, National Sun Yat-sen University, Kaohsiung City, Taiwan; 5 Department of Psychiatry, Tri-Service General Hospital Beitou Branch, National Defense Medical Center, Taipei City, Taiwan; 6 Graduate Institute of Aerospace and Undersea Medicine, National Defense Medical Center, Taipei City, Taiwan; University of Lausanne, SWITZERLAND

## Abstract

**Background:**

Aircrew members are required to attend hypoxia awareness training regularly to strengthen their memory of their personal hypoxia symptoms by undergoing training inside a hypobaric chamber. The aim of this study was to examine the association between hypoxia symptoms experienced during two training sessions that were 4 years apart.

**Methods:**

This was a crossover study to compare hypoxia symptoms and self-reported physiological effects of trapped gas between a previous training session and a current training session in an altitude chamber. The subjects were military crew members who undertook a 25,000-feet refresher training course in 2018. We used a structured questionnaire to obtain the target information before and during hypoxia exposure. Data were analyzed using SPSS software.

**Results:**

A total of 341 trainees participated in this survey and completely filled out the questionnaire. Gastrointestinal tract discomfort caused by the expansion of trapped gas was the main physiological reaction during the previous and current training sessions. Frequently reported symptoms were poor concentration (30.5%), impaired cognitive function (20.5%), visual disturbances (16.4%), hot flashes (15.8%), and paresthesia (12.6%) during both exposures. However, the proportions of participants reporting poor concentration (*P* = 0.378) and visual disturbances (*P* = 0.594) were not significantly different between the recalled and current training sessions. The five most common symptoms among the subjects with less than 1,000 flight hours were poor concentration (29.8%), visual disturbance (27.3%), impaired cognitive function (14.9%), dizziness/lightheadedness (11.6%), and hot flashes (9.9%), which overlapped substantially with the symptoms reported by other subjects. The occurrence of those five most common symptoms in the group with more than 1,000 flight hours did not significantly differ between the recalled training session and the current training session.

**Conclusions:**

The most common hypoxia symptoms reported were similar between the recalled and current training sessions in an environment with a low oxygen concentration. This finding was also clearly affected by the duration of flight experience. Moreover, GI effects of the expansion of trapped gas were commonly observed at low atmospheric pressure.

## Introduction

Oxygen is necessary to maintain survival and normal body function. There is a constant 20.95% oxygen in the air. At sea level, the atmospheric pressure is approximately 760 mmHg, and the partial pressure of oxygen is 160 mmHg. However, as altitude increases, the total atmospheric pressure decreases, resulting in a decrease in the partial pressure of oxygen due to the presence of fewer molecules per unit volume [1].

In-flight hypoxia has been recognized as a threat to human performance and flight safety, even with the development of supplemental oxygen and cabin pressurization. During their aviation careers, approximately 15% of fighter pilots suffer from in-flight hypoxia [2]. From previous findings, it appears that the main causes of hypoxia-related incidents are cabin depressurization, oxygen system failure, hose disconnection, unsealed masks, mask removal, and physiological conditions, among others [3, 4]. According to a United States Air Force (USAF) survey on the health effects of depressurization events in military aircraft, hypoxia was reported in 63.1% of the cases and barotrauma in 20% [5].

Pilots exposed to low oxygen concentrations show some objective signs and experience some subjective symptoms of hypoxia over time. Starting from the onset of hypoxia, there is a time window called the time of useful consciousness (TUC), which depends on the flight altitude, during which the pilots can take measures to correct the hypoxia. This time allows pilots to effectively initiate recovery procedures before being incapacitated. The average TUC value at resting state is 3 to 5 minutes at 25,000 feet [6]. The TUC sharply decreases to only a few seconds at higher altitudes. Aircrew members must be able to detect hypoxia at an early stage based on previous experience. The symptoms of hypoxia are classified into five categories: impairment of cognitive function, visual changes, psychomotor impairment, symptoms of psychological disturbances, and nonspecific symptoms [7, 8]. Because hypoxia symptoms vary across individuals, pilots must experience such effects to improve their ability to rapidly respond to them and thus prevent hypoxic damage.

In 1941, the United States Navy (USN) started to use hypobaric chambers to simulate high-altitude flight environments and provide pilots with physiological training in such conditions. Trainees must remember the unique personal symptoms they experience during induced hypoxia. In addition, they have to acquire the essential skills to correctly operate the on-board oxygen supply system to avoid hypoxic accidents and to cope with the physiological issues induced by the change in air pressure [9]. In most countries, refresher training every 3–5 years is mandatory to maintain accurate memories [10].

An analysis showed that there were 656 in-flight hypoxia incidents in the USAF from 1976 to 1990 [11]. Of these, 606 involved hypobaric chamber-trained aircrew members, and only 3.8% of the pilots experienced loss of consciousness. Of the 50 untrained pilots involved, 94% experienced loss of consciousness. This study showed that 26.2% of the trained crew recognized their own symptoms based on the symptoms they had experienced in the chamber. In other studies, the pilots were able to recognize ongoing in-flight hypoxia events due to failed pressurization based on their symptoms and the experience gained during hypobaric training [4, 5]. Formal reports collecting and comparing trainees’ experiences and physiological reactions before and after chamber flights have been rare. Thus, we investigated the association of hypoxia symptoms recalled from a prior chamber flight and reported during a current chamber flight. We also examined the incidence of various physiological experiences due to trapped gas in the analyzed aircrew members in the recalled experience and the recent exposure.

## Materials and methods

### Design

In accordance with the Manual of Military Aviation Medicine in Taiwan, tri-service military aircrew members must attend an initial hypobaric chamber flight, followed by refresher hypoxia awareness training sessions in the hypobaric chamber every four years. The most important goals for the subjects are as follows: (1) to acquaint themselves with the physiological effects of atmospheric pressure changes; (2) to practice techniques to resolve the consequent physical discomfort; (3) to recognize and familiarize themselves with their personal hypoxia symptoms; and (4) to learn and apply the appropriate skills to prevent hypoxia-induced incapacitation.

In this context, our study was performed to obtain information regarding the experience of hypoxia during prior chamber training and acute exposure symptoms experienced by trainees during ongoing chamber flights. We conducted this crossover study in 2018 to investigate the associations between symptoms. All trainees had to pass an annual health examination and obtain clearance to attend the chamber flight training from the squadron’s flight surgeon.

### Equipment

Hypoxia awareness training was conducted with the hypobaric chamber Contract 540 (Guardite Inc., Chicago, IL). We designed a structured questionnaire to collect information about the experience of hypobaric hypoxia during prior chamber training session and the current chamber flight [2–4, 12]. The questionnaire consisted of three parts: part I, demographic data (age, sex, role, flight years, flight hours); part II, self-reported physiological discomfort induced by atmospheric change (ear blockage, sinus blockage, trapped gas in the gastrointestinal [GI] tract, tooth pain); and part III, symptoms of hypoxia recognized during training, such as poor concentration, hot flashes, impaired cognitive function, dizziness/lightheadedness, visual disturbances, numbness, air hunger, paresthesia, fatigue, anxiety, tingling, and nausea.

### Protocol

We recruited military aircrew members who underwent a 25,000-feet refresher chamber flight in the Aviation Physiology Research Laboratory (APRL) between January 1 and December 31, 2018. During the prechamber flight briefing, the APRL instructor obtained informed consent from the trainees and administered the questionnaires to collect the data on their memory of a past chamber flight. Moreover, the instructor explained the purpose of and procedures involved in the flight, as well as the need to perform ear and sinus checks in all participants.

**Fig 1:**
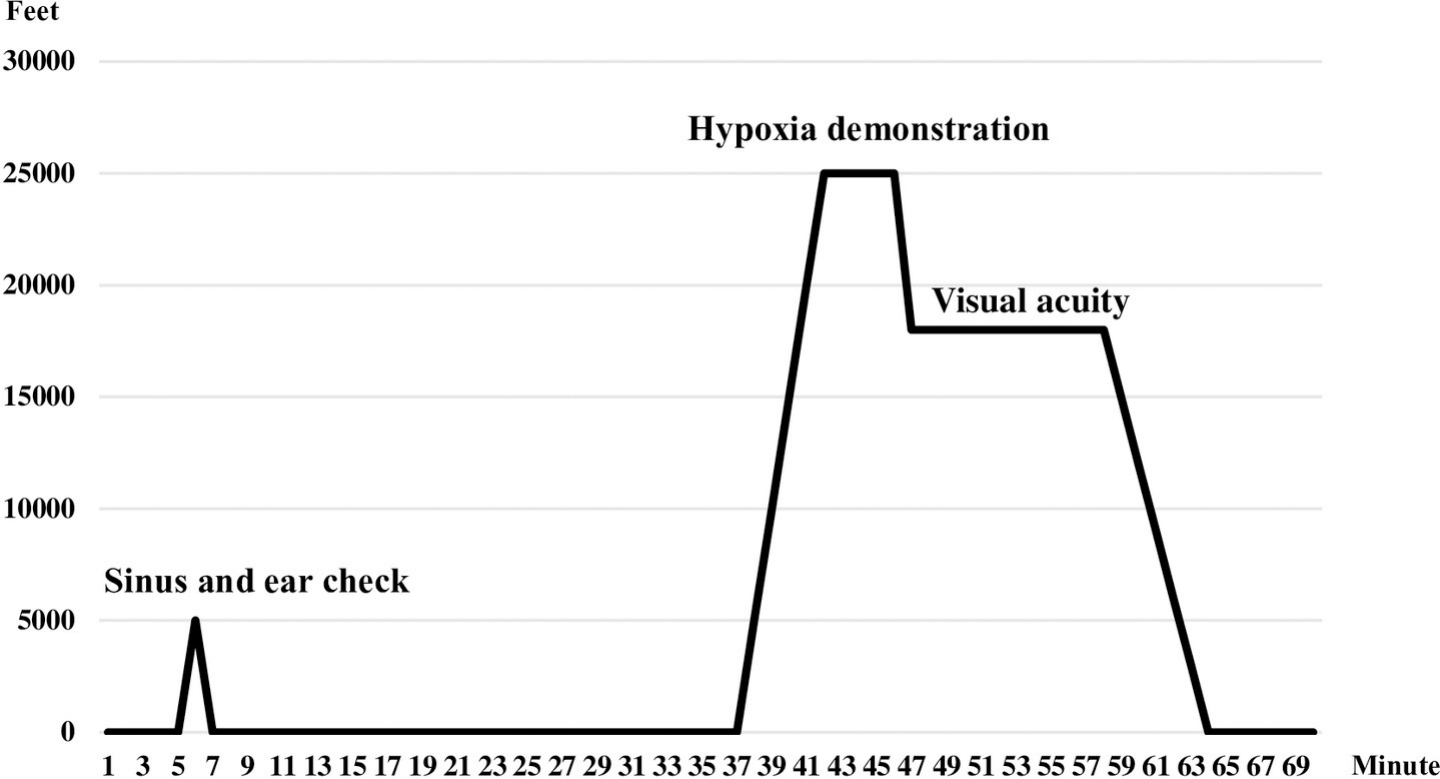
Chamber flight profile.

The chamber flight began with a sinus check at 5,000 feet and 30 minutes of denitrogenation; then, the simulated altitude ascended to 25,000 feet at a rate of 5,000 feet per minute. The hypoxia demonstration was conducted at 25,000 feet, and the effects of hypoxia on visual acuity were tested at an altitude of 18,000 feet. The chamber altitude then returned to ground level to complete the training (Fig 1). Immediately after exiting the chamber, the participants reported their hypoxia symptoms on the questionnaire. Three hundred forty-seven participants volunteered to participate in this survey. Six of them were excluded because of incomplete data on the remembered chamber flight. Finally, three hundred forty-one participants completed the questionnaires and were entered into the analysis.

### Ethics approval

This work was approved by the Institutional Review Board of Kaohsiung Armed Forces General Hospital in Kaohsiung City, Taiwan (No. KAFGH 107–017).

### Statistical analyses

For the descriptive analysis, the means ± standard deviations were used to describe the distributions of continuous variables. Categorical data are shown as numbers and proportions. The consistency of the correlation of hypoxia symptoms between the remembered chamber flight and the current chamber flight was examined using the McNemar test. All data were analyzed using SPSS software, version 24 (IBM, Armonk, NY). A two-tailed *P* value <0.05 was considered statistically significant.

## Results

Of the 341 participants, the average age, flight years, and flight hours were 35.8±7.2 years, 11.8±7.7 years, and 1156.1±1134.3 hours, respectively. Of all participants, 331 (97.1%) were male and 288 (84.5%) were pilots, as shown in Table 1.

**Table 1:** Demographic data of study participants (*n* = 341).

Variables	Mean ± SD*/n* (%)
Age, years	35.8±7.2
<30	70 (20.5)
30–39	163 (47.8)
40–49	89 (26.1)
≥50	19 (5.6)
Sex	
Male	331 (97.1)
Female	10 (2.9)
Role	
Pilot	288 (84.5)
Nonpilot	53 (15.5)
Flight years	11.8±7.7
<5	93 (27.3)
5–9	61 (17.9)
10–19	131 (38.4)
≥20	56 (16.4)
Flight hours	1156.1±1134.3
<500	72 (21.1)
500–999	49 (14.4)
1,000–1,999	106 (31.1)
≥2,000	114 (33.4)

SD: standard deviation

Table 2 summarizes the physiological events due to trapped gas effects experienced by the trainees during the chamber flight. In the recalled training session, the incidence rates of trapped gas in the GI tract, sinus blockage, and ear blockage were 7.3%, 4.1%, and 2.3%, respectively. Trapped gas in the GI tract was also the main event reported after the current training session. The incidence of trapped gas-related problems were higher in the current training session than in the recalled training session. The reproducibility of these symptoms was not high, as only a few pilots experienced the same trapped gas-related symptoms 4 years apart.

**Table 2:** Comparison of trapped gas-related physiological events (*n* = 341).

Variables	Recalled training session	Current training session	Both (*n*)	*P* value^¶^
	*n* (%)	*n* (%)	*n* (%)	
Ear blockage	8 (2.3)	47 (13.8)	1 (0.3)	<0.001
Sinus blockage	14 (4.1)	16 (4.7)	3 (0.9)	0.839
Trapped gas in the GI tract	25 (7.3)	52 (15.2)	15 (4.4)	<0.001
Tooth pain	3 (0.9)	1 (0.3)	0 (0.0)	NA

GI: gastrointestinal; NA: not applicable

^¶^: Comparison between remembered and current chamber flights performed with the McNemar test

As shown in Table 3, the subjects reported their symptoms before and after hypoxia awareness training. Before the training, the five most frequently noted hypoxia symptoms were poor concentration (47.2%), impaired cognitive function (40.5%), visual disturbances (28.4%), hot flashes (27.6%), and paresthesia/numbness (21.7%). After the training, the most commonly mentioned symptoms were poor concentration (44.3%), impaired cognitive function (32.6%), visual disturbances (30.2%), and hot flashes (38.4%). Notably, nearly 10% of the trainees reported experiencing no symptoms during the recalled experience and the current training. Of the 12 symptoms, the five leading symptoms that subjects experienced during both the recalled and current training sessions were poor concentration (30.5%), impaired cognitive function (20.5%), visual disturbances (16.4%), hot flashes (15.8%), and paresthesia (12.6%). The occurrence rates of poor concentration and visual disturbances were not significantly different between the recalled experience and the current session.

**Table 3:** Comparison of self-reported symptoms during hypoxia awareness training (*n* = 341).

Symptoms	Recalled training session	Current training session	Both	*P* value^¶^
	*n* (%)	*n* (%)	*n* (%)	
Poor concentration	161 (47.2)	151 (44.3)	104 (30.5)	0.378
Hot flashes	94 (27.6)	131 (38.4)	54 (15.8)	0.001
Impaired cognitive function	138 (40.5)	111 (32.6)	70 (20.5)	0.012
Dizziness/lightheadedness	57 (16.7)	107 (31.4)	36 (10.6)	<0.001
Visual disturbances	97 (28.4)	103 (30.2)	56 (16.4)	0.594
Numbness	74 (21.4)	101 (29.6)	32 (9.4)	0.010
Air hunger	52 (15.2)	95 (27.9)	35 (10.3)	<0.001
Paresthesia	74 (21.7)	93 (27.3)	43 (12.6)	0.045
Fatigue	68 (19.9)	84 (24.6)	39 (11.4)	0.081
Anxiety	28 (8.2)	55 (16.1)	18 (5.3)	<0.001
Tingling	44 (12.9)	32 (9.4)	14 (4.1)	0.111
Nausea	11 (3.2)	10 (2.9)	4 (1.2)	0.999
Other	24 (7.0)	7 (2.1)	3 (1.0)	0.001
No symptoms	37 (10.9)	26 (7.6)	11 (3.2)	0.117

^¶^: Comparisons between the recalled and current training sessions were made with the McNemar test

After stratification by flight hours, as shown in Table 4, the five most common symptoms among the subjects with less than 1,000 flight hours were poor concentration (29.8%), visual disturbances (27.3%), impaired cognitive function (14.9%), dizziness/lightheadedness (11.6%), and hot flashes (9.9%) in both hypoxia awareness training sessions. Those symptoms substantially overlapped with those reported by the subjects with more than 1,000 flight hours. In contrast to the group with less than 1,000 flight hours, however, the incidence of the main symptoms in the group with more than 1,000 flight hours was not significantly different between the recalled and current training sessions.

**Table 4:** Comparison of main self-reported symptoms during hypoxia awareness training stratified by flight hours (*n* = 341).

Symptoms	Recalled training session	Current training session	Both	*P* value^¶^
			*n* (%)	
	*n* (%)	*n* (%)		
< 1000 hours (*n* = 121)				
Poor concentration	56 (46.3)	56 (46.3)	36 (29.8)	1.000
Hot flashes	25 (20.7)	45 (37.2)	12 (9.9)	0.005
Impaired cognitive function	45 (37.2)	34 (28.1)	18 (14.9)	0.126
Dizziness/lightheadedness	22 (18.2)	41 (33.9)	14 (11.6)	0.002
Visual disturbances	20 (16.5)	33 (27.3)	33 (27.3)	0.029
Paresthesia	13 (10.7)	20 (16.5)	5 (4.1)	0.210
≥ 1000 hours (*n* = 220)				
Poor concentration	105 (47.7)	95 (43.2)	68 (30.9)	0.260
Hot flashes	69 (31.4)	86 (39.1)	42 (19.1)	0.057
Impaired cognitive function	93 (42.3)	77 (35.0)	52 (23.6)	0.064
Dizziness/lightheadedness	35 (15.9)	66 (30.0)	22 (10.0)	<0.001
Visual disturbances	77 (35.0)	70 (31.8)	45 (20.5)	0.427
Paresthesia	61 (27.7)	73 (33.2)	38 (17.3)	0.148

^¶:^ Comparisons between the recalled and current training sessions were made with the McNemar test

## Discussion

To our knowledge, our study is one of the few to discuss the trapped gas-related physiological effects that occur during hypobaric exposure. Our results showed that those physiological reactions were experienced by one-third of the trainees during a refresher training session. We also discovered some similarities between the recalled hypoxia symptoms and those reported immediately after a training session, especially among subjects with more flight hours.

Vargo *et al*. reviewed the historical training records of the United States Army hypobaric chamber exposure from 2014 to 2016. Most physiological events were categorized as issues involving trapped gas during exposed to a hypobaric environments. Therefore, our investigation focused on the physiological effects caused by the barometric change. The results of the United States Army study showed that among minor physiological symptoms, trainees identified trapped gas and dysbarism of the ears, teeth, or sinuses as their main complaints, which was in line with our findings [12]. In fact, ear or sinus pain was a common reason to interrupt the training while descending. Based on the grading scale of chamber reactions, all severe physiological events during chamber flight were recorded on the worksheet by trained observers [13]. However, our study additionally assessed the number of GI problems and detected a fair number of events. A potential explanation is that no case met the safety reporting criteria indicating that they needed to be treated or that the chamber flight need to be interrupted during the study period. Therefore, the questionnaire could be used to gather data on mild physiological effects not recorded on the worksheet. In the literature review, we did not find studies that investigated the relationship between physiological events experienced in different chamber flights. We additionally compared the incidences of symptoms of trapped gas between the recalled training session and the current training session. Only sinus blockage was not significantly different between the two sessions. There was a low incidence of problems related to gas expansion. This might be related to the physiological condition of the subjects during the chamber flight.

At high altitudes, hypoxia causes aircrew members to have insufficient alveolar oxygen and a lower partial pressure of oxygen. This could affect a pilot’s performance and is thus recognized as one of the life-threatening hazards in aviation. In their study, Temme *et al*. found that the subjects experienced a 53% decrease in the control ability necessary to maintain the flight simulator at the target airspeed, altitude, and heading [14]. Hypobaric chamber training has been the classic method of demonstrating the effects of hypoxia on aircrew members for many years. There are several reports in the literature showing that aircrew members who have been trained in hypoxic conditions are able to respond more efficiently to in-flight hypoxic accidents [4, 5, 11]. Hypoxia symptoms seem to vary across individuals in terms of intensity, speed of onset, and order of appearance. However, researchers have concluded that, the hypoxia symptoms experienced by each individual are likely to remain the same over time. For military personnel, the purpose of hypoxia awareness training in hypobaric chambers is to learn to recognize their unique “hypoxia signature” [7]. The memory of that individual hypoxia signature may gradually diminish over time. Therefore, repeated exposures at intervals of 3 to 6 years are performed to reinforce individuals’ knowledge of their own hypoxia symptoms, facilitating their recall of those symptoms during an emergency [15].

In the USAF refresher physiology course, Woodrow *et al*. reported that the most frequent symptoms experienced during hypoxia training were dizziness, lightheadedness, mental confusion, tingling, and visual impairment [16]. A survey conducted in Saudi Arabia indicated that the common symptoms experienced during hypoxia awareness training included poor concentration, confusion, a slowing of the response time, and a perceived reduction in the color/light intensity [7]. In New Zealand, Johnston *et al*. found that symptoms, including cognitive impairment, visual changes, lightheadedness/dizziness, lack of coordination, and slurred speech, often reappeared during subsequent hypoxia training sessions [15]. The main symptoms reported in previous studies overlap with those identified in our work: mental problems, dizziness/lightheadedness, and visual changes, identified in our work. In summary, the symptoms mentioned above are sensitive indicators of hypoxia exposure across training sessions. Woodrow et al. showed that some participants experienced no symptoms during the previous or current training session [16]. Similarly, in this study, a small portion of trainees did not describe experiencing any hypoxia symptoms during the recalled training session or the current training session. Individuals need to become familiar with the symptoms of hypoxia to facilitate its early detection and correction by military aircrew members to avoid incapacitation. We could not eliminate the possibility that they recovered before the onset of hypoxia symptoms and prevented the loss of consciousness. In addition, subjective recall and reporting errors might exist, which could have led to the underestimation of the prevalence of hypoxia symptoms.

Our findings showed that of the twelve symptoms considered, half had higher reported frequencies during the current training session than during the recalled training session. Then, there were significant differences in the occurrences of two-thirds of symptoms between the two chamber flights. This might indicate that the memory of hypoxia symptoms fades over time and varies among individuals. Because the goal of the refresher training is to enhance trainees’ recognition of their personal symptoms within a certain time interval, the number of chamber flights experienced should be related to the memory of hypoxia symptoms. Theoretically, the number of hypoxic awareness training sessions is positively associated with the duration of flight experience. We further categorized participants into different groups based on their duration of flying experience. The results showed that the dominant symptoms in each subgroup were in line with those in former reports [2, 4, 7, 17]. Among senior aircrew members, however, the main symptoms described during the recalled and current training sessions were not different. Thus, this finding suggests that the memory of hypoxia symptoms could be strengthened by repeated training sessions.

The reviews on aircraft hazards and accidents from the USN, USAF, and the Royal Australian Air Force show that the majority of in-flight hypoxia situations have occurred when fighter pilots wore their oxygen masks and used oxygen equipment [2–4]. The pilots did not recognize the danger until it was too late. However, the chamber training protocol requires that participants remove their masks and breathe the ambient air at the set altitude. They are exposed to a hypoxic environment and perform a variety of tests. When they detect hypoxia symptoms, they immediately take corrective action by inhaling 100% oxygen. The “mask-off” signal is a warning sign given to make trainees aware of the beginning of the hypoxia demonstration inside the hypobaric chamber, and such a signal is not given during a real in-flight emergency situation. Furthermore, decompression sickness during low-pressure exposure is still a concern, even if oxygen prebreathing can minimize the risk [18–20]. A “mask-on” training strategy would be worth introducing during hypoxia awareness training under normobaric conditions.

The Naval Aerospace Medical Research Laboratory developed reduced oxygen breathing devices (ROBD) to be used for hypoxia training under normal pressure. The ROBD hypoxia induction mechanism controls the percentage of nitrogen in the air to simulate altitude exposure with the regulator [2]. Researchers found that there was no significant difference in the hypoxic scenarios induced by a ROBD and a hypobaric chamber [21, 22]. To address the shortcomings of the simulation of atmospheric changes, novel training techniques were implemented that combined the features of the hypobaric chamber and the ROBD in Australia. The model was named Combined Altitude and Depleted Oxygen (CADO). During CADO training, the subjects were exposed to a pressure altitude of 10,000 feet in a hypobaric chamber while simultaneously breathing air with a low oxygen concentration to induce hypoxic symptoms [23]. Additionally, in this case, all relevant hypoxia symptoms experienced by the test subjects did not differ when induced by CADO or the hypobaric chamber. An additional benefit of this strategy is that not only is there a low risk of adverse physiological impacts but also a “mask-on” training method could be adopted based on the combination of ROBD and CADO training. To date, neither training method is available in Taiwan. We suggest that the APRL establish the effectiveness of ROBD and CADO training and recommend the appropriate hypoxic exposure methods.

### Limitations

Our study had some limitations. First, we used a questionnaire to gain insight into the subjective symptoms of previous hypoxia exposure. Recall or report errors could not be completely excluded from this study. Second, percentages were used to describe the frequency of experiencing each symptom in our work. However, the fact that the order of appearance of the symptoms varied among individuals might have influenced the incidence rates. An alternative method of calculating the severity score should be considered [23, 24]. Third, common symptoms could have been emphasized by instructors during training or by the questionnaire itself. Fourth, demographic and physical characteristics might have influenced the frequency of hypoxia symptoms. Although the results were stratified by the duration of flight experience, residual confounders could still exist in this study. Next, since our goal was to clarify the biological reactions to acute hypoxia exposure, we only recruited trainees participating in a chamber flight at 25,000 feet. However, some reports have indicated that helicopter pilots also experience issues with hypoxia during flights without supplemental oxygen [24, 25]. Aircrew members undertaking an 18,000-feet refresher training session in Taiwan were excluded from this study. Finally, no in-flight details on hypoxia were available due to administrative restrictions. In the future, we will extend this work to compare symptoms between chamber training session hypoxia and in-flight hypoxia.

## Conclusions

In conclusion, we found that the main hypoxia symptoms include poor concentration, visual disturbances, impaired cognitive function, dizziness/lightheadedness, and hot flashes during previous and current chamber flights. Especially for senior aircrew, the symptoms were consistent between the recalled training session and the current training session. Trapped gas in the GI tract was the most common physiological reaction induced by the hypobaric environment during the training.
